# Differences in larval survival and IgG response patterns in long-lasting infections by *Trichinella spiralis*, *Trichinella britovi* and *Trichinella pseudospiralis* in pigs

**DOI:** 10.1186/s13071-020-04394-7

**Published:** 2020-10-16

**Authors:** Edoardo Pozio, Giuseppe Merialdi, Elio Licata, Giacinto Della Casa, Massimo Fabiani, Marco Amati, Simona Cherchi, Mattia Ramini, Valerio Faeti, Maria Interisano, Alessandra Ludovisi, Gianluca Rugna, Gianluca Marucci, Daniele Tonanzi, Maria Angeles Gómez-Morales

**Affiliations:** 1 Department of Infectious Diseases, Istituto Superiore di Sanità, viale Regina Elena 299, 00161 Rome, Italy; 2 Istituto Zooprofilattico Sperimentale of Lombardy and Emilia Romagna, via Pietro Fiorini 5, 40127 Bologna, Italy; 3 Department of Public Health, Azienda Unità Sanitaria Locale of Modena, Strada Martiniana 21, 4112 Baggiovara, Modena Italy; 4 Consiglio per la ricerca in agricoltura e l’analisi dell’economia agraria, Centro di Ricerca Zootecnica e Acquacoltura, Sede di Modena, via Beccastecca 345, 41018 S. Cesario sul Panaro (MO), Italy

**Keywords:** *Trichinella spiralis*, *Trichinella britovi*, *Trichinella pseudospiralis*, Pig, Larval burden, Immune response, ELISA, Western blot, Epidemiology

## Abstract

**Background:**

Domesticated and wild swine play an important role as reservoir hosts of *Trichinella* spp. and a source of infection for humans. Little is known about the survival of *Trichinella* larvae in muscles and the duration of anti-*Trichinella* antibodies in pigs with long-lasting infections.

**Methods:**

Sixty pigs were divided into three groups of 20 animals and infected with 10,000 larvae of *Trichinella spiralis*, *Trichinella britovi* or *Trichinella pseudospiralis*. Four pigs from each group were sacrificed at 2, 6, 12, 18 and 24 months post-infection (p.i.) and the number of larvae per gram (LPG) of muscles was calculated. Serum samples were tested by ELISA and western blot using excretory/secretory (ES) and crude antigens.

**Results:**

*Trichinella spiralis* showed the highest infectivity and immunogenicity in pigs and larvae survived in pig muscles for up to 2 years p.i. In these pigs, the IgG level significantly increased at 30 days p.i. and reached a peak at about 60 days p.i., remaining stable until the end of the experiment. In *T. britovi-*infected pigs, LPG was about 70 times lower than for *T. spiralis* at 2 months p.i. and only very few infecting larvae were detected at 6 months p.i., whereas no larvae were detected at 12, 18 and 24 months p.i. At 6 months p.i., degenerated/calcified larvae and cysts were detected in the muscles by trichinoscopy and histology. The IgG pattern showed by *T. britovi*-infected pigs was similar to that of *T. spiralis*-infected pigs, although seroconversion occurred some days later. The larval burden of *T. pseudospiralis* was slightly greater than for *T. britovi* at 2 months p.i., but no larvae were detected at 6 and 12 months p.i. In *T. pseudospiralis*-infected pigs, seroconversion occurred slowly, as in *T. britovi*-infected pigs. The IgG level showed a significant drop at 6 months p.i. and declining to the cut-off value at 12 months p.i.

**Conclusions:**

The longer survival of *T. spiralis* in pigs in comparison with the other two species highlights its exceptional dissemination potential. These results provide an explanation of the controversial data collected by parasitological and serological tools in the course of epidemiological investigations.
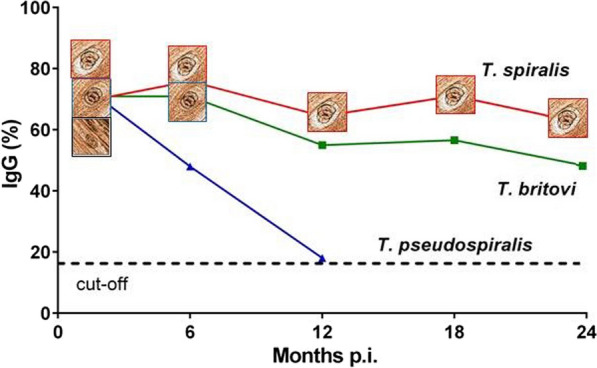

## Background

The history of the biology and epidemiology of parasites of the genus *Trichinella* has been strongly linked to swine since the first discovery of this nematode in a naturally infected animal [1]. After this first report and in the following 154 years, pork and pork-derived products from domestic and wild swine have represented and still represent the main source of trichinellosis infections for humans [2, 3]. From the mid-19th century up to recent years, there have been numerous investigations on the biology and epidemiology of these zoonotic nematodes in swine. Of the 13 known taxa of the genus *Trichinella*, as many as seven (*Trichinella spiralis*, *T. nativa*, *T. britovi*, *T. pseudospiralis*, *T. nelsoni*, *T. papuae* and *T. zimbabwensis*) have been detected in naturally or experimentally infected swine, with varying levels of both infectivity and persistence [4–10]. For this reason, the domestic pig has been the subject of numerous studies since the 19th century [11, 12].

According to the international literature, *T. spiralis* is the species most frequently detected in domestic and wild swine (*Sus scrofa*), followed by *T. britovi* and *T. pseudospiralis* [13–18]. Extensive experimental investigations have demonstrated a different larval burden and immune response for *Trichinella* species in swine at 60 [8] and 70 [7] days post-infection and up to 40 weeks [6]. No systematic information is available on the survival of *Trichinella* larvae and the presence of detectable anti-*Trichinella* IgG in pigs experimentally infected by different *Trichinella* species for longer time periods.

Epidemiological investigations carried out in several world regions to determine *Trichinella* prevalence by artificial digestion of muscle tissues and/or by serology in domestic and wild swine have revealed a discrepancy between parasitological and serological positivity [19–30]. This inconsistency has created uncertainty as to the specificity and/or sensitivity of the tests and the consequent epidemiological value of the results.

The aim of this study was to evaluate the infectivity and larval persistence in muscle of *T. spiralis*, *T. britovi* and *T. pseudospiralis* in experimentally infected pigs tested for a two-year period. The persistence of detectable antibodies and their relationship with larva recovery were also studied, as these factors provide important epidemiological information.

## Methods

### Study design, pigs and *Trichinella* spp. strains

A batch of 60 homogenous (Duroc × Large White) pigs, 15 weeks of age, 35 kg, was divided into 3 groups of 20 pigs each and infected with 10,000 larvae as follows: group 1, *T. spiralis* (ISS3, domestic pig, Poland); group 2, *T. britovi* (ISS2, red fox, *Vulpes vulpes*, Italy); and group 3, *T. pseudospiralis* (ISS13, raccoon, *Procyon lotor*, Russia). Previously, these *Trichinella* strains had been maintained by serial passages in CD1 Swiss mice for more than 20 years, never in pigs before this study. Each group of 20 pigs was housed in a separate fenced enclosure.

Ten ml of blood was collected from the jugular vein of each pig 10 days before infection, on the day of infection (time 0), and once a month until slaughter. The sera were isolated from clotted blood by centrifugation and then frozen at − 20 °C. Based on previous investigations [6, 8], blood samples were also collected during the expected seroconversion period from pigs in the three groups at 24 days post-infection (p.i.) (six *T. spiralis-*, five *T. britovi-* and five *T. pseudospiralis*-infected pigs), 35 days p.i. (all 60 pigs) and 42 days p.i. (five pigs: two infected with *T. britovi* and three with *T. pseudospiralis*, that had been seronegative at 35 days p.i.). Four pigs in each group/*Trichinella* species were killed at 2, 6, 12, 18 and 24 months p.i. Each pig was weighed before slaughter, and mean ± standard deviation (SD) weights were: 59 ± 5.5 kg at 2 months p.i., 139.2 ± 15.3 kg at 6 months p.i., 144 ± 20.9 kg at 12 months p.i., 198.5 ± 27.6 kg at 18 months p.i., and 241.9 ± 18 kg at 24 months p.i. Then pigs were sedated with 1 ml/20 kg of Azaperone, stunned with a captive-bolt, immediately jugulated and bled. The blood was collected in trays measuring about 80 × 50 cm so that it could clot and enable the collection of good quality serum. Each carcass was eviscerated, and the diaphragm and tongue were removed and weighed separately. The carcass was then cut lengthwise. The following cuts or muscles were dissected and weighed: diaphragm; tongue; masseter; neck; front (shoulder muscles) and hind (ham muscles) legs; loin; and ribs. For large muscles, part of the muscle nearest to the tendons was collected. About 150 g of each muscle or group of muscles was cut into fragments measuring about 2 × 2 cm and preserved in plastic containers with labels stating the pig code number, muscle name, and slaughter date. Muscle samples were preserved at 4 °C for 24–48 h and then frozen at − 20 °C until digestion.

The pigs were housed in the Animal Care Unit of the Centro di Ricerca Zootecnica e Acquacoltura of San Cesario sul Panaro (Modena, Italy) according to European Directive 63/2010/EU.

### Detection of *Trichinella* spp. larvae in pig muscles

One day before artificial digestion, the muscle samples were thawed at 4 °C. One-hundred grams of each muscle, without fat and fascia, was digested using the reference method applied by the European Commission [31]. For *T. pseudospiralis* infected muscles, the digest of muscle samples of *T. pseudospiralis* infected pigs were immediately cooled on ice and allowed to settle for 20 min to stop the digestion process. Larvae were counted in triplicate under a stereomicroscope at 40× magnification.

### Detection of *Trichinella* spp. larvae in pig muscles by trichinoscopy and histology, reproductive capacity index in mice, and larva identification

Within 24–48 h after slaughter, 100 g of the diaphragm of one pig from each of the three pig groups was digested as previously described and the larvae were washed, counted and injected *per os* into 2 mice (500 larvae each), to assess the reproductive capacity index (RCI, number of larvae collected/number of larvae injected). Ten larvae from each pig were stored in 90% ethanol for *Trichinella* species identification by multiplex PCR [32]. Furthermore, 100 small pieces of oat-grain-sized diaphragm samples, collected from *T. britovi*- and *T. pseudospiralis*-infected pigs at 6 months p.i., were compressed between two thick glass plates until they became translucent and were examined individually to detect *Trichinella* larvae using a stereomicroscope at 15–40× magnification. The muscle tissue fragments containing a collagen cyst with or without larva were collected from the compressorium and fixed in 10% formalin. Tissue samples were embedded in paraffin and the histological section obtained from the paraffin blocks were Giemsa stained and examined under a light microscope.

### Serology

The anti-*Trichinella* IgG from each individual serum were tested by excretory/secretory antigen (ES) ELISA, ES western blot (Wb), crude worm extract (CWE) ELISA and CWE-Wb according to previous published protocols [33–35]. Briefly, the pig sera were diluted 1:50 and 1:100 for ELISA and Wb, respectively. Peroxidase-labelled anti-swine IgG was diluted 1:30,000 (Kierkegaard and Perry Laboratories, Gaithersburg, MD, USA). The optical density (OD) was obtained by reading the reaction at 450 nm using an ELISA plate microtiter reader (Dynex Technologies, Chantilly, VA, USA). For each serum sample, an ELISA index (E_I_) expressed as a percentage of positivity was calculated according to Gómez-Morales et al. [34]. Positive control serum samples were from *Trichinella* infected pigs, whether naturally or experimentally infected, in which *Trichinella* larvae had been detected by artificial digestion and their sera had been tested positive by ELISA (OD range 1.000–2.870). The cut-off value, calculated as the mean ± 3 SD of the OD values of the 880 serum samples from *Trichinella*-free pigs, was 18% (Additional file 1: Figure S1) [34].

The Wb analysis was carried out according to Gómez-Morales et al. [33, 35]. Briefly, 150 μg of total proteins corresponding to *T. spiralis* ES antigens or CWE was diluted and loaded into 10% pre-cast NuPage Novex Bis-Tris Gels® (Life Technologies, Carlsbad, CA, USA) as described in the instructions for electrophoresis using the XCell SureLock® Mini-Cell (Life Technologies). Proteins were separated using electrophoresis under reducing conditions and transferred to nitrocellulose (Bio-Rad, Hercules, CA, USA) at room temperature (RT) for 1 h. The nitrocellulose filters were blocked with 5% skimmed milk in 1× Tris Borate Saline Tween (TBST, 50 mM Tris pH 8.0, 150 mM NaCl, 1% Tween 20) at 4 °C overnight and washed 3 times with 1× TBST. The nitrocellulose filters were cut into strips, each of which was then incubated with swine sera with 3% w/v skimmed milk (Sigma-Aldrich, Saint Louis, USA) in 1× TBST at RT for 1 h. After washing 3 times with 1× TBST, the strips were incubated for 1 h with a 1:3000 dilution of goat anti-pig IgG conjugated with horseradish peroxidase (Bio-Rad).

### Statistical analysis

The analysis considered larva concentrations as the number of larvae per gram (LPG) of muscle or group of muscles. For each *Trichinella* species, the number of LPG within each muscle or group of muscles was described using the mean with SD. Changes in mean LPG between two consecutive time-points were evaluated using the t-test. Differences in mean LPG among muscles or groups of muscles at different time-points were also evaluated by repeated measures analysis of variance (ANOVA), using the Bonferroni correction for pairwise comparisons. The rate of decrease in mean LPG between two consecutive time-points (T_0_ and T_1_) due to an increase in muscle weight and a reduction in the number of larvae was estimated. The expected mean LPG at T_1_ in the absence of a reduction in LPG as E_1_ = N_0_/W_1_ (where N_0_ is the LPG at T_0_ and W_1_ is the muscle weight at T_1_) was calculated. The rate of decrease in mean LPG due to a reduction in the number of larvae was then calculated as E_1_ / (µ_0_ − µ_1_), where µ_0_ and µ_1_ indicate the mean LPG at T_0_ and the mean LPG at T_1_, respectively. The rate of decrease in mean LPG due to an increase in muscle weight was calculated as 1 − E_1_ / (µ_0_ − µ_1_).

For each *Trichinella* species, the trend of E_I_ over time was evaluated using mixed regression models to account for dependency of observations among the individual pigs. E_I_ was also compared among species at different time-points using ANOVA models with Bonferroni correction for pairwise comparisons. Finally, the relationship between LPG and the level of anti-*Trichinella* IgG by species, muscle or muscle group and time-point was evaluated using Pearson’s correlation coefficient.

Statistical significance was set at *P* < 0.05. Non-parametric tests were used to evaluate differences in mean LPG and E_I_ between two or more groups of pigs (i.e. Wilcoxon rank-sum test and Kruskal-Wallis test). Results from parametric and non-parametric analyses led to the same conclusions, except in a very few cases analysed by the Wilcoxon rank-sum test. The analyses were performed using Stata/SE version 16.0 (StataCorp LLC, Texas, USA).

## Results

One pig in the *T. spiralis* group died 2 weeks after infection and the animal was deleted from the trial. All the other *T. spiralis*- and *T. britovi*-infected pigs were killed at the established times according to the experimental design because larvae were present in the muscles and/or because of persistent positive anti-*Trichinella* IgG responses. *T. pseudospiralis*-infected pigs were killed at 2 and 6 months p.i. according to the experimental design. All the other pigs (*n* = 12) were killed at 12 months p.i. due to the disappearance of larvae in the muscles of pigs killed 6 months before and the pronounced reduction in the IgG level below the cut-off in 8 out of 12 pigs. In the other 4 pigs, the E_I_ values were still above the cut-off (29.6%; 41.9%; 25.3%; 24.6%) at 12 months p.i.

### Larval burden

**Fig. 1:**
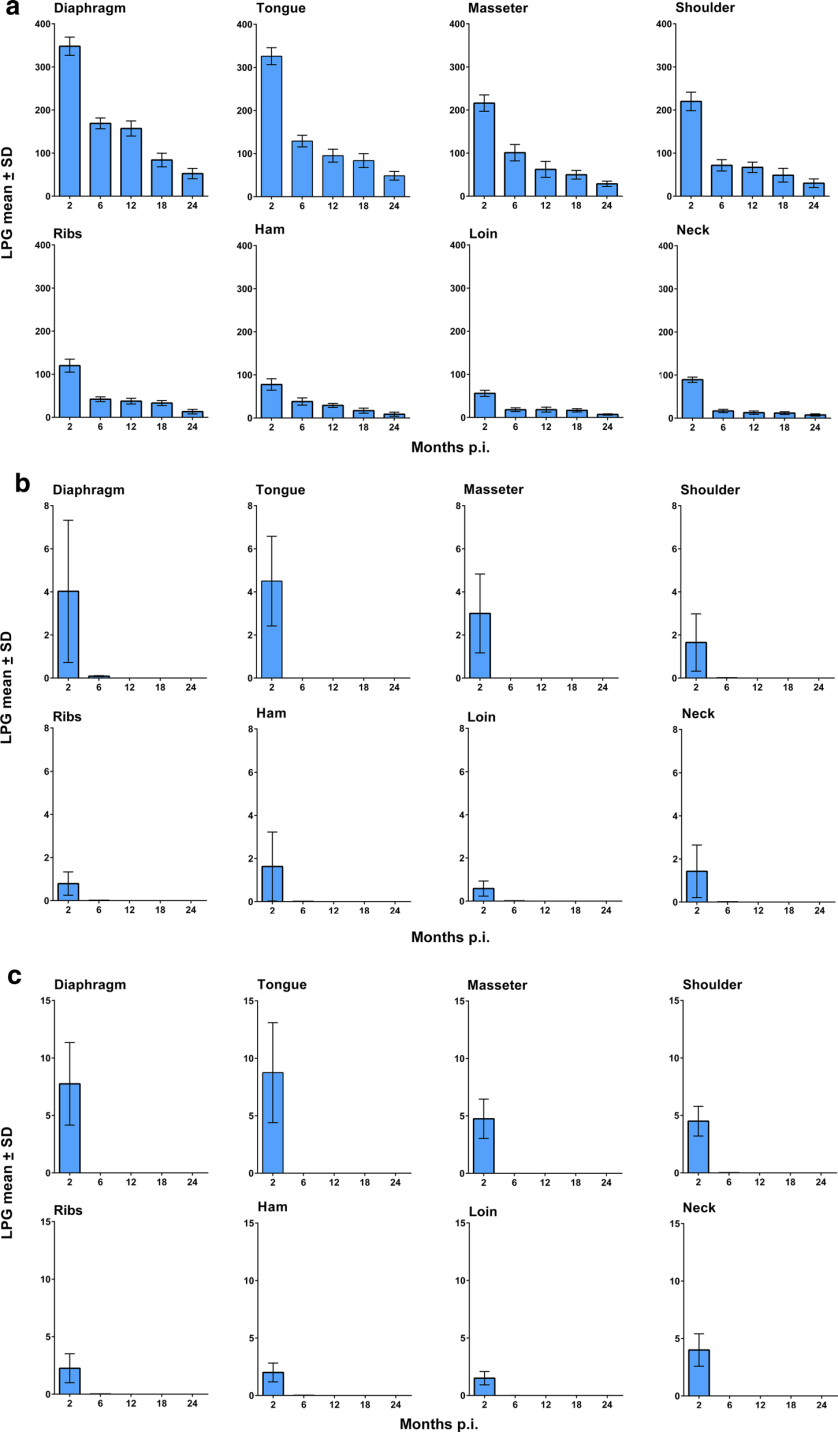
Mean number (± standard deviation, SD) of muscle larvae per g (LPG) in muscles or groups of muscles of *Trichinella spiralis* (**a**), *Trichinella britovi* (**b**) and *Trichinella pseudospiralis* (**c**) experimentally infected pigs at different months post-infection (p.i.)

At two months p.i., *T. spiralis* showed the highest larval burden per g of muscle in all muscles compared to the other two species (Fig. 1a; Additional file 2: Table S1). The highest larval burden was detected in the diaphragm (348 LPG, 95% CI: 324–373) followed by the tongue (326 LPG, 95% CI: 303–349), masseter (216 LPG, 95% CI: 194–238) and shoulder (220 LPG, 95% CI: 195–245), ribs (120 LPG, 95% CI: 102–138), ham (77.7 LPG, 95% CI: 62.2–93.2), neck (89.3 LPG, 95% CI: 82.2–96.5) and loin (56.0 LPG, 95% CI: 47.8–64.2). The variability of larval burden among the eight investigated muscle sites at 2, 6, 12, 18 and 24 months p.i. is shown in Fig. 1a and the statistically significant pairwise comparison in Additional file 2: Table S1.

*Trichinella britovi* showed the lowest larval burden in the eight investigated muscle sites compared to *T. spiralis* and *T. pseudospiralis* two months p.i. At this time p.i., the LPG in the diaphragm and tongue was 82 and 72 times lower than that of *T. spiralis*, respectively. At two months p.i., the highest larval burden was detected in the tongue (4.50 LPG, 95% CI: 2.40–6.60) followed by the diaphragm (4.25 LPG, 95% CI: 0.92–7.6), masseter (3.00 LPG, 95% CI: 1.16–4.84), shoulder (1.65 LPG, 95% CI: 0.30–2.99), ham (1.63 LPG, 95% CI: 0.00–3.31), neck (1.43 LPG, 95% CI: 0.19–2.66), ribs (0.79 LPG, 95% CI: 0.24–1.34) and loin (0.58 LPG, 95% CI: 0.22–0.94). At six months p.i., no larvae were detected in the tongue and masseter of the 4 pigs tested, and very few larvae were detected in the other six muscle sites of one pig and in the diaphragm of another. No larvae of *T. britovi* were detected by artificial digestion in any of the 8 investigated muscle sites at 12, 18 and 24 months p.i. (Fig. 1b; Additional file 2: Table S1).

The larval burden for *T. pseudospiralis* was slightly higher than for *T. britovi* at two months p.i. At this time-point, the LPG in the diaphragm and tongue was 45 and 37 times lower than for *T. spiralis*, respectively. At two months p.i., the highest larval burden was detected in the tongue (8.75 LPG, 95% CI: 4.36–13.2), followed by the diaphragm (7.75 LPG, 95% CI: 4.12–11.4), masseter (4.75 LPG, 95% CI: 3.03–6.47), shoulder (4.50 LPG, 95% CI: 3.20–5.80), neck (4.00 LPG, 95% CI: 2.57–5.43), ribs (2.25 LPG, 95% CI: 0.98–3.52), ham (2.0 LPG, 95% CI: 1.18–2.82) and loin (1.50 LPG, 95% CI: 0.92–2.08). At six months p.i., no larvae were detected in any of the eight investigated muscle sites in 4 pigs. At 12 months p.i., no larvae were detected in any of the other 12 pigs.

**Fig. 2:**
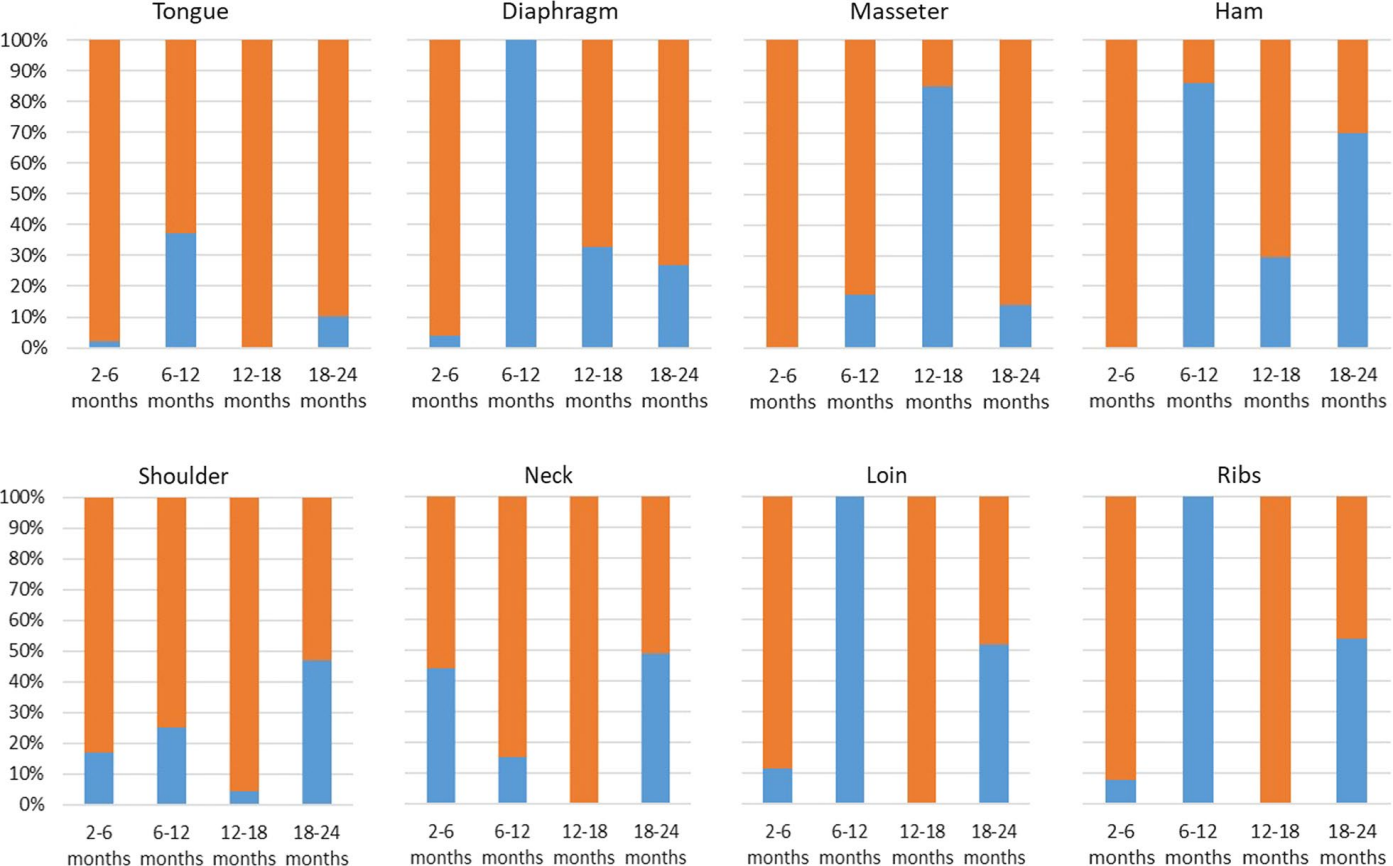
Percentage decrease in mean of *Trichinella* spp. muscle larvae per gram due to increased weight of muscles (orange) or larval reduction in muscles (blue) at different months post-infection

At six months p.i., the larval burden for *T. spiralis* showed a significant reduction in LPG in all of the eight muscles or groups of muscles tested (4.84 ≤ *t*_(5)_ ≤ 19.6, *P* < 0.005). The reduction in larval burden was due to muscle growth and larval degeneration (Fig. 2). The proportion of these two factors reducing the larval burden varied according to the muscle or group of muscles and the time-point p.i. Muscle growth was the main factor in the reduced larval burden in all of the muscles tested and, particularly, in the tongue, masseter, shoulder, neck and loin at 6, 12, 18 and 24 months p.i. Conversely, the degeneration process followed by larval death played a primary role in the reduced larval burden in the diaphragm, ham, loin and ribs between 6 and 12 months p.i. and in the masseter between 12 and 18 months p.i. (Fig. 2).

**Fig. 3:**
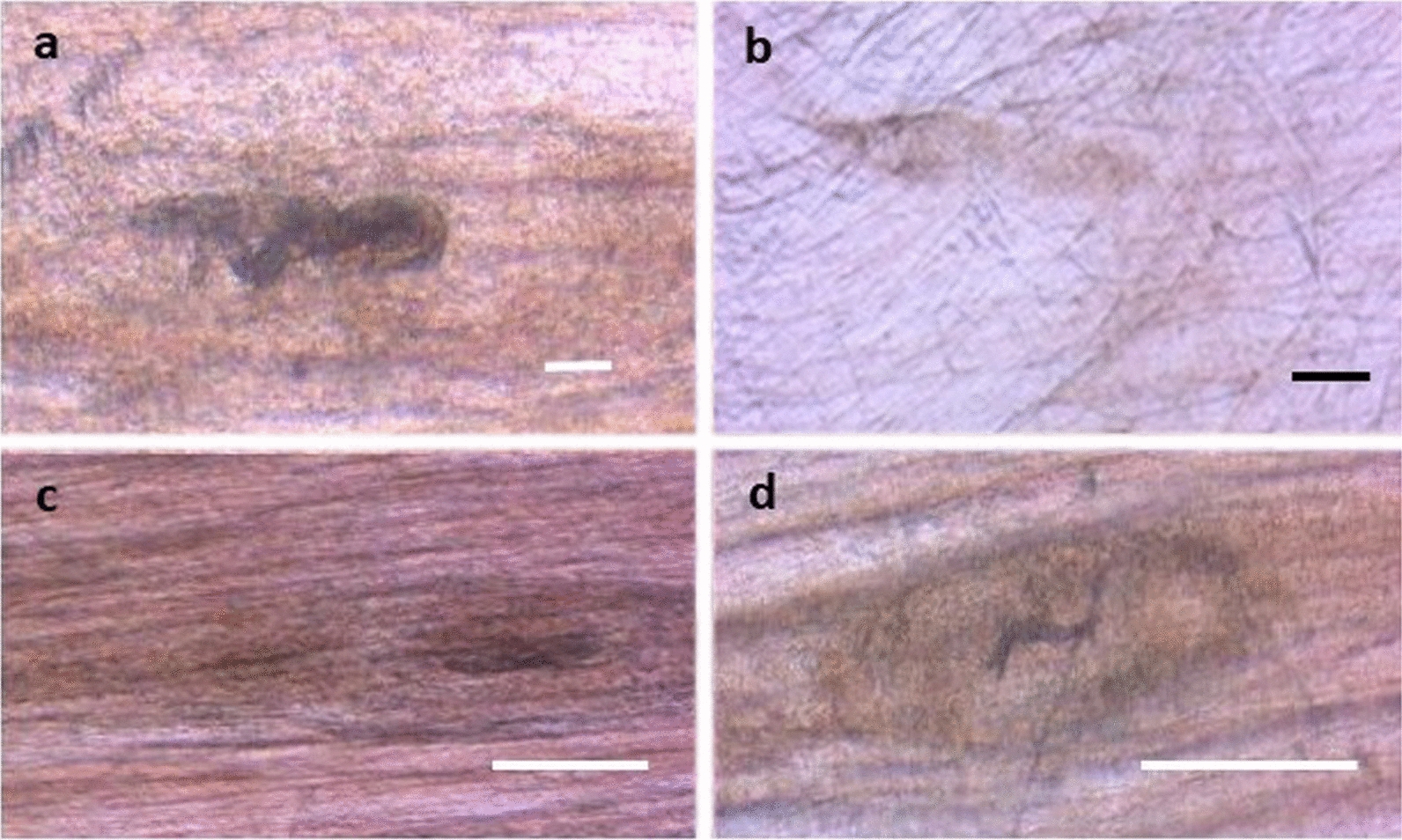
*Trichinella britovi* nurse cell-larva complexes in a pig diaphragm by compressorium, six months post-infection. *Scale-bars*: **a**, 50 µm; **b**, **c**, **d**, 200 µm

**Fig. 4:**
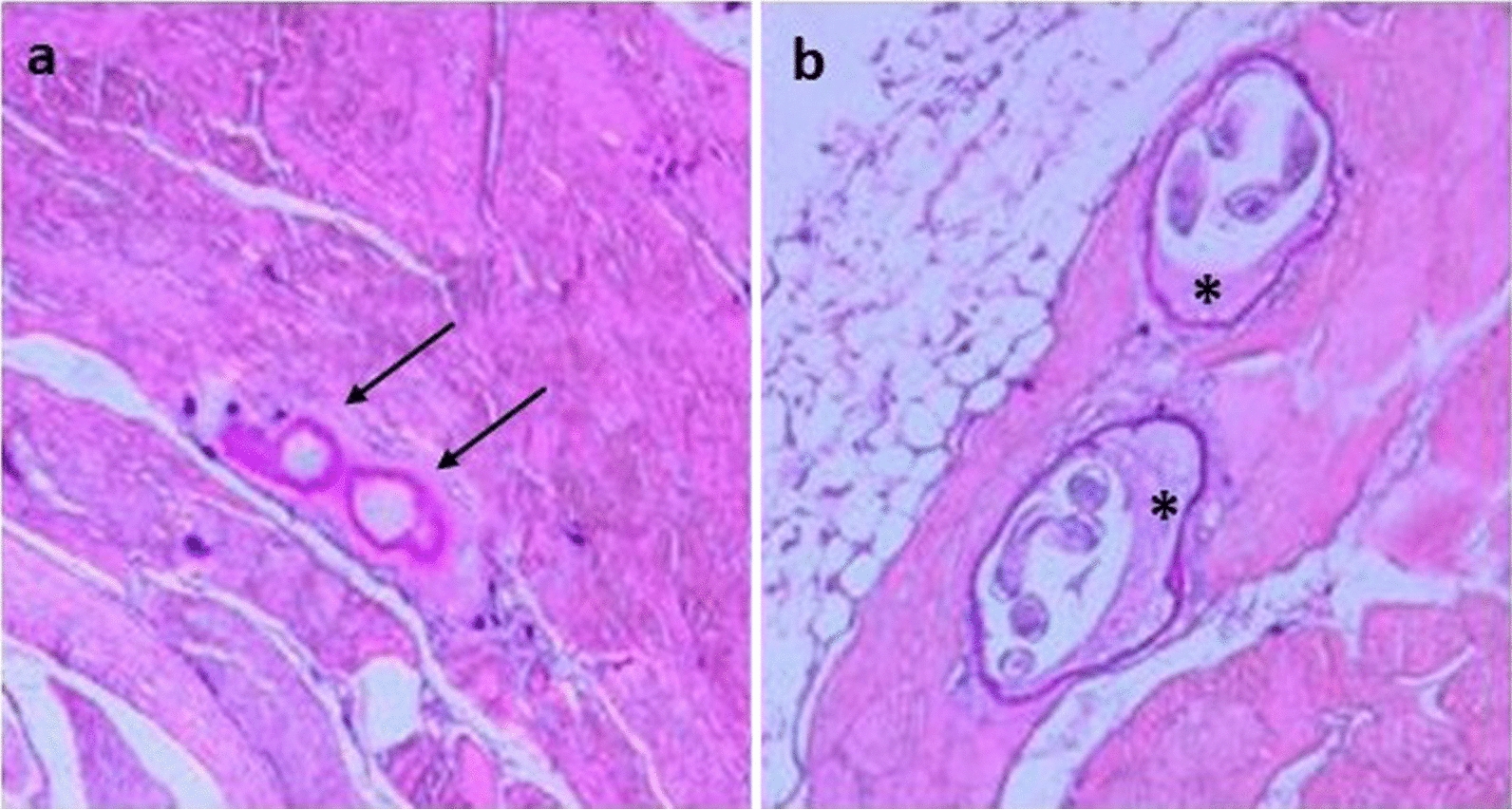
Histology of *Trichinella britovi* nurse cell-larva complexes in a pig diaphragm, six months post-infection: empty cysts (arrows) at original magnification 50× (**a**); and cysts with fibrosis (asterisks) and larva inside at original magnification 100× (**b**)

At six months p.i., no *T. britovi* larvae were detected in the tongue and masseter, whereas some larvae were detected in the other six muscles or groups of muscles in the four pigs tested using digestion. The compressorium and histological examinations of diaphragm samples from a *T. britovi*-infected pig showed degenerated cysts with dead larvae (Fig. 3) and emptied cysts and cysts with larva with evident fibrosis (Fig. 4). It should be noted that no inflammatory cells were present around the nurse-cell-larva complexes (Fig. 4). No degenerated/calcified larvae were detected in the diaphragms of *T. pseudospiralis*-infected pigs by compressorium or histology at six months p.i. (data not shown).

### Infectivity of larvae from pig muscles in mice

*Trichinella spiralis* larvae collected from the diaphragm showed an average RCI of 67 in CD1 mice, without significant statistical differences at 2, 6, 12, 18 and 24 months p.i. (data not shown). *Trichinella britovi* and *T. pseudospiralis* larvae collected from the diaphragm at two months p.i. showed an average RCI of 27 and 31 in CD1 mice, respectively. *Trichinella britovi* larvae detected in pig muscles at six months p.i. were very motile, suggesting infectivity, but there were too few of them to infect mice and thus calculate the RCI.

All 10 larvae collected from the diaphragms of *T. spiralis-*, *T. britovi*- and *T. pseudospiralis*-infected pigs at all time-points p.i. were correctly identified as belonging to the *Trichinella* species of the inoculum (data not shown).

### Immune response

**Fig. 5:**
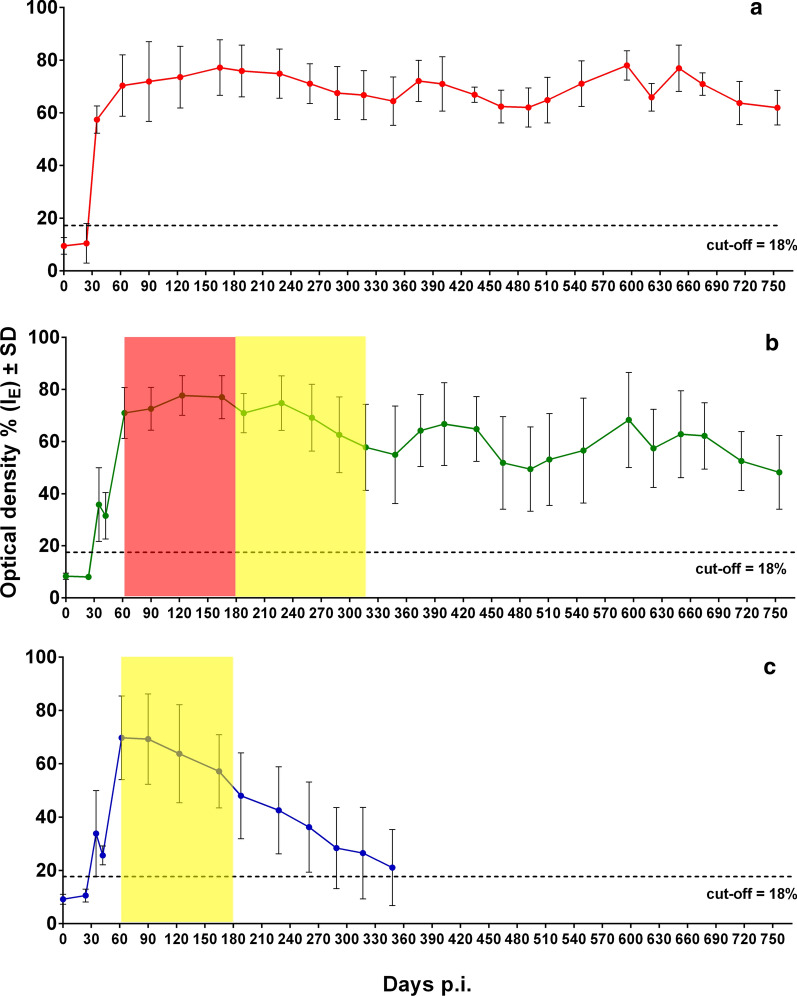
Anti-*Trichinella*-IgG patterns by an excretory/secretory *T. spiralis* antigen-ELISA in pigs infected with *Trichinella spiralis* (**a**), *Trichinella britovi* (**b**), and *Trichinella pseudospiralis* (**c**). The red area in **b** shows the period of time of a high reduction of the larval burden in the muscles. The yellow area in **b** and **c** shows the period of time elapsed from the last finding of larvae in the muscles of the four pigs tested to the following monitoring, in which no larvae were found in the muscles of the four pigs tested

No statistically significant difference was observed between E_I_ values detected by ES and crude antigens. Of 6 *T. spiralis*-infected pig sera tested 24 days post-infection (p.i.), only one was positive by ELISA. At 35 days p.i., all 19 *T. spiralis*-infected pig sera tested positive by ELISA. The E_I_ values then reached a plateau (around 70% E_I_) and remained stable until the end of the experimental period of just over two years, without any statistically significant trend over time (regression coefficient (β) = 0.001, *P* = 0.784) (Fig. 5a).

Of 5 *T. britovi*-infected pig sera tested at 24 days p.i., there were no positive results by ELISA. At 35 days p.i., serum samples from 18 (90%) *T. britovi*-infected pigs were positive by ELISA, and at 42 days p.i., the sera from the other two previously negative pigs had seroconverted. The E_I_ values then increased up to an average of 70% (60 days p.i.), which persisted up to 300 days p.i. From that point onwards, the average E_I_ value was around 60% until the end of the experiment, showing a slight statistically significant decrease over time (β = -0.019, *P* = 0.001) (Fig. 5b).

Of 5 *T. pseudospiralis*-infected pig sera tested at 24 days p.i., there were no positive results by ELISA. At 35 days p.i., serum samples from 17 (85%) *T. pseudospiralis*-infected pigs were positive by ELISA, and at 42 days p.i., the sera from the other 3 previously negative pigs had seroconverted. The E_I_ values then increased up to an average of 70% at 60 days p.i. Afterwards, the E_I_ values showed a prominent statistically significant decrease (β = -0.093, *P* < 0.001) up to 12 months p.i., when the mean of E_I_ values fell below the cut-off (Fig. 5c).

The comparison of the specific IgG levels among the three species revealed that at one month p.i., *T. spiralis* induced a higher level of anti-*Trichinella* IgG than *T. britovi* and *T. pseudospiralis* (*F*_(2, 56)_ = 20.06, *P* < 0.001), whereas at 6 months p.i. (*F*_(2, 45)_ = 25.72, *P* < 0.001) and 12 months p.i. (*F*_(2, 33)_ = 29.36, *P* < 0.001) there was a significant decrease in the specific IgG level in *T. pseudospiralis*-infected pigs (Fig. 5, Table 1). The correlation between the number of LPG and anti-*Trichinella* IgG by species, muscle/group of muscles and time-points is shown in Table 2. At two months p.i., there was a positive significant correlation between the number of LPG recovered from the diaphragm of pigs infected with *T. pseudospiralis* and the level of specific IgG (*r* = 0.99, *P* < 0.001), whereas at the same time p.i., the correlation between both parameters was significantly negative in the loin muscle (*r* = -0.99, *P* < 0.001). A significant positive correlation was found for *T. britovi*-infected pigs only at 6 months p.i. (*r* = 0.95, *P* < 0.001). For *T. spiralis*, no significant correlation of LPG was found, either by muscle or by time-point. The overall correlation between the number of LPG and anti-*Trichinella* IgG, without distinction by species, muscle/muscle group or time-point, was not significant (*r* = 0.27, *P* > 0.05).

**Table 1:** Comparison of anti-*Trichinella* IgG (ELISA index) mean among *Trichinella spiralis*, *Trichinella britovi* and *Trichinella pseudospiralis* at different times post-infection

Time (months p.i.)	Mean anti-*Trichinella* IgG (SD)			*P*-value^a^	Statistically significant pairwise comparison (Bonferroni adjusted)
	*T. spiralis*	*T. britovi*	*T. pseudospiralis*		
1	57.4 (5.2)	35.8 (14.1)	33.8 (16.1)	< 0.001	*T. spiralis vs T. britovi* and *T. pseudospiralis*
2	70.3 (11.7)	71.0 (9.8)	69.7 (15.7)	ns	
6	75.9 (9.8)	70.9 (7.5)	48.0 (16.1)	< 0.001	*T. pseudospiralis vs T. spiralis* and *T. britovi*
12	64.5 (9.2)	54.9 (18.7)	21.0 (14.3)	< 0.001	*T. pseudospiralis vs T. spiralis* and *T. britovi*
18	71.1 (8.7)	56.6 (20.2)	na	ns	
24	61.9 (6.6)	48.2 (14.1)	na	ns	

^a^ANOVA model

*Abbreviations*: na, not applicable; ns, not statistically significant (*P* > 0.05)

**Table 2:** Correlation between larvae per gram and anti-*Trichinella* IgG (ELISA index) by species, muscle/group of muscles and time post-infection

Time (months p.i.)	Tongue	Diaphragm	Masseter	Ham	Shoulder	Neck	Loin	Ribs
*T. spiralis*								
2	0.592	− 0.172	0.649	0.138	− 0.240	− 0.800	− 0.304	− 0.56
6	0.070	− 0.726	0.260	− 0.052	− 0.631	− 0.897	− 0.805	− 0.76
12	− 0.484	− 0.132	− 0.584	0.231	− 0.245	− 0.636	− 0.027	− 0.232
18	0.681	− 0.565	− 0.028	0.278	0.423	0.570	− 0.175	− 0.643
24	0.709	0.604	0.076	− 0.515	0.384	− 0.223	0.432	0.298
*T. britovi*								
2	0.362	0.496	0.777	0.227	0.739	0.531	0.820	0.527
6	na	0.954*	na	0.532	0.532	0.532	0.532	0.532
*T. pseudospiralis*								
2	0.991*	− 0.671	− 0.274	− 0.126	0.944	0.388	− 0.990*	− 0.783
All species								
2	0.510	0.489	0.510	0.493	0.490	0.490	0.479	0.467

^*^Statistically significant (*P <* 0.05)

*Abbreviation*: na, not applicable

The western blot pattern of serum samples collected from *T. spiralis*- and *T. britovi*-infected pigs at 1, 2, 12 and 24 months p.i. showed the triple-band diagnostic pattern, irrespective of the presence (*T. spiralis*) or absence (*T. britovi*) of larvae in the muscles at 12 and 24 months p.i. The pattern of serum samples collected from *T. pseudospiralis*-infected pigs at 2, 6 and 9 months p.i. showed the triple-band diagnostic pattern, irrespective of the presence (at 2 months p.i.) or absence (at 6 and 9 months p.i.) of larvae in the muscles (data not shown).

## Discussion

The experimental infection of pigs showed three species-specific parasitological and immunological patterns. *Trichinella spiralis* larvae survived in the striated muscles of pigs for two years, with a seven-fold reduction in LPG in the diaphragm from two to 24 months p.i. (Fig. 1a). In all *T. spiralis*-infected pigs, seroconversion occurred within 35 days p.i., rapidly reached a plateau of about 70% E_I_ at 62 days p.i., and then remained constant throughout the experimental period (Fig. 5a). *Trichinella britovi* larvae survived in the striated muscles of pigs for less than six months and very few motile larvae were detected in the diaphragm of one pig and in the diaphragm and five other muscle groups of another pig of the four animals tested at six months p.i. (Fig. 1b). The number of LPG in the diaphragm was 42 times lower than that detected at two months p.i. (Additional file 2: Table S1). Seroconversion occurred in all but two infected animals within 35 days p.i., reached an E_I_ value of about 70% at two months p.i. and then decreased after eight months to an E_I_ value of about 60%. This value remained quite stable up to 23 months p.i., when the E_I_ value declined to 50% at 24 months p.i. (Fig. 5b). *Trichinella pseudospiralis* larvae survived in the striated muscles of pigs for less than six months (Fig. 1c). Seroconversion occurred slowly, reached a peak of 70% E_I_ at about 60 days p.i. and then rapidly decreased to around the cut-off after ten months (Fig. 5c). These three different biological and immunological patterns are in agreement with those found in previous investigations, which explored the parasitological and immunological patterns over a shorter period of time [6–8] and provide an explanation of the epidemiology of these three parasites.

The western blot triple-band diagnostic pattern of sera from *T. spiralis-*, *T. britovi*- and *T. pseudospiralis*-infected pigs did not change from two to 24 months p.i. (12 months p.i. for *T. pseudospiralis*). It follows that this pattern does not reveal the presence or absence of infecting larvae in the pig muscles, which would have been very useful for sero-epidemiological investigations.

According to an unpublished study, *T. spiralis* larvae survived and retained their infectivity in sow muscles for three years, but no data were recorded on detectable anti-*Trichinella* IgG in sera from these sows (K. D. Murrell, personal communication). In another study, *T. spiralis* larvae and anti-*Trichinella* IgG were detected in a pig infected with 5000 larvae 130 weeks earlier [36].

The detection of anti-*Trichinella* IgG in *T. britovi*-infected pigs at 24 months p.i. (Fig. 5b) in which viable larvae had been detected only 18 months earlier, can be explained by the immunogenicity of this parasite species and the persistence of the humoral immune responses due to the continuous release of *Trichinella* antigens from the encysted larvae before to become degenerated/calcified. This hypothesis is supported by studies showing that humoral immune responses against *T. britovi* in humans can still be detected 15 years after infection [37, 38]. In *T. pseudospiralis*-infected pigs, the shorter period of survival of larvae in the muscles compared to *T. britovi*, the lack of a collagen capsule around the larva and the lower immunogenicity of the parasite in comparison with *T. spiralis* and *T. britovi* [39, 40] could explain the more rapid decrease in antibodies (Fig. 5c), given that no live larvae or degenerated/calcified larvae were detected at six months p.i.

The role of the swine race and *Trichinella* spp. strains in infection patterns has been investigated by several authors. At a high infection dose (20,000 larvae), *T. britovi* (ISS100) and *T. pseudospiralis* (ISS013) showed higher infectivity in Iberian pigs than German Yorkshire SPF pigs. However, no differences were observed at lower infecting doses (200 and 1000 larvae) [8]. *Trichinella pseudospiralis* strains from Europe (ISS013), North America (ISS470) and Australia (ISS141) showed different infection patterns in pigs (York × Duroc) and wild boar (*S. scrofa*). The infection patterns for *T. spiralis* (ISS004) and *T. britovi* (ISS100) were similar in both pigs (York × Duroc) and wild boar [6, 7]. All of these three studies concluded that *T. spiralis* is highly infective and *T. britovi* and *T. pseudospiralis* are moderately infective in swine. The results of this study are in agreement with these previous studies and highlight the *T. spiralis-*swine relationship, which dates back to the Pliocene epoch [41, 42].

According to the International Trichinella Reference Centre (EP, unpublished data), *T. spiralis* has been detected in 1153 (80.9%) domestic pigs and 1468 (55.7%) wild boar, *T. britovi* in 270 (18.9%) domestic pigs and 1119 (42.4%) wild boar, and *T. pseudospiralis* in two (0.1%) domestic pigs and 49 (1.8%) wild boar. These data confirm the results of epidemiological investigations carried out in Europe, where these three *Trichinella* species coexist [15–18, 43–51]. The longer survival of *T. spiralis* in domestic pigs compared to the other two species highlights the exceptional dissemination potential conferred on *T. spiralis* by its association with domesticated swine and the maintenance of the domestic cycle. In Europe, backyard pigs are slaughtered in the wintertime and pork scraps from slaughter and uncooked pork scraps from the kitchen are inappropriately used to feed other pigs present in the backyard, which will be slaughtered in subsequent years. Conversely, the occurrence of *T. britovi* and, to a lesser extent, of *T. pseudospiralis* in domestic pigs can be explained by the practice used by hunters of feeding domestic pigs with leftovers from hunted wild boar or other wild animals in the hunting season, which precedes the slaughter of pigs by a few weeks and thus leaves just enough time for *T. britovi* and, to a lesser degree, *T. pseudospiralis* to develop in pig muscles. In central and southern European countries, where the prevalent species among wildlife is *T. britovi*, epidemiological investigations carried out following human outbreaks caused by the consumption of pork and/or pork-derived products from backyard or free-range pigs have in all cases shown a relationship with hunting activities [13, 52–56].

Epidemiological investigations on feral pigs in the USA carried out using serology and artificial digestion followed by larval species identification have shown a serological and parasitological prevalence of 2.9% and 1.81%, respectively, and all larvae were identified as *T. spiralis* [28]. In a similar study carried out on wild boar in Italy, the serological and parasitological prevalence was 2.2% and 0.007%, respectively, and all larvae were identified as *T. britovi* [27]. These epidemiological data are congruent with the results of this study. In fact, in the USA, where *T. spiralis* circulates among feral pigs, the serological prevalence was only 1.6 times higher than the parasitological prevalence. By contrast, in Italy, where *T. britovi* circulates among wild boar, the serological prevalence was 314 times higher than the parasitological prevalence due to the short survival period of *T. britovi* larvae in the muscles and the more lengthy persistence of anti-*T. britovi* IgG.

## Conclusions

The results presented here contribute to our understanding of the controversial data collected by parasitological and serological tools in the course of epidemiological investigations. The correlation between the presence of larvae in muscles and of anti-*Trichinella* IgG in serum samples for a time frame of at least two years, can be used for epidemiological purposes only in the regions where *T. spiralis* is the predominant species in domestic and/or wild swine. In these regions, serum epidemiological investigations will not only provide indications on host-parasite contacts but, with a certain approximation, also information on the number of infected animals.

## Supplementary information

**Additional file 1: Figure S1.** Scatter plot of the ELISA index (EI) for anti-*Trichinella* spp. IgG in *Trichinella* free pigs from different European countries. ELISA was preformed using excretory/secretory antigens.

**Additional file 2: Table S1.** Mean number, standard deviation (SD) and 95% confidence interval (CI) of larvae per g (LPG) of *Trichinella spiralis*, *Trichinella britovi* and *Trichinella pseudospiralis* in experimentally infected pigs at different times post infection.

## Data Availability

All data generated or analysed during this study are included in the article and its additional files.

## Abbreviations

ANOVA: Analysis of variance
CI: Confidence intervals
CWE: Crude worm extract
E/S: Excretory/secretory
E_I_: ELISA index
ELISA: Enzyme-linked immunosorbent assay
IgG: Immunoglobulin G
LPG: Larvae per gram
OD: Optical density
p.i.: Post-infection
PCR: Polymerase chain reaction
r: Regression coefficient
RT: Room temperature
SD: Standard deviation
TBST: Tween-Tris Buffered Saline
Wb: Western blot
