# Supplementary figures and images for: Differences in larval survival and IgG response patterns in long-lasting infections by Trichinella spiralis, Trichinella britovi and Trichinella pseudospiralis in pigs
Source: Parasit Vectors. 2020 Oct 16;13:520. doi: 10.1186/s13071-020-04394-7 (PMC7566126; doi:10.1186/s13071-020-04394-7)

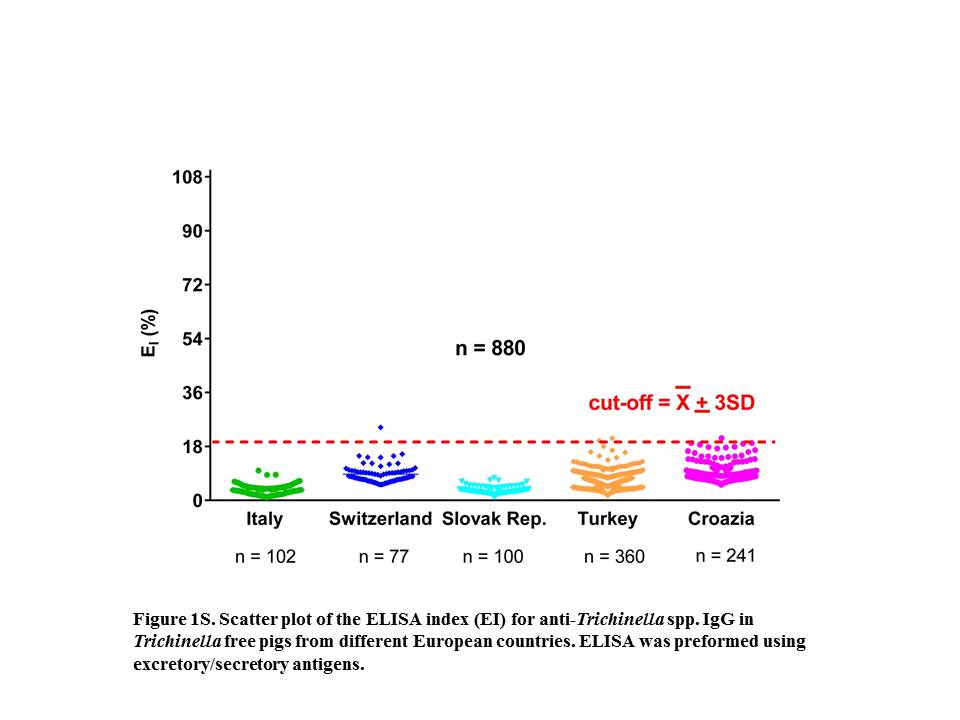

Supplement: Supplementary file 1 — Additional file 1: Figure S1. Scatter plot of the ELISA index (EI) for anti-Trichinella spp. IgG in Trichinella free pigs from different European countries. ELISA was preformed using excretory/secretory antigens. [file 13071_2020_4394_MOESM1_ESM.jpg]
